# Targeting fibrosis in the treatment of lower urinary tract dysfunction

**DOI:** 10.1002/path.70079

**Published:** 2026-05-27

**Authors:** Ajinkya R Limkar, Sophia M Vrba, Emily A Ricke, Zsuzsanna Fabry, Matthew S Lee, Kevin T McVary, William A Ricke

**Affiliations:** ^1^ Department of Urology, George M. O'Brien Center for Research Excellence University of Wisconsin Madison Madison WI USA; ^2^ Medical Scientist Training Program University of Wisconsin Madison Madison WI USA; ^3^ Department of Pathology and Laboratory Medicine University of Wisconsin Madison Madison WI USA; ^4^ Department of Urology, Wexner Medical Center The Ohio State University Columbus OH USA; ^5^ Loyola University Stritch School of Medicine Maywood IL USA; ^6^ Center for Male Health Loyola University Medical Center Maywood Maywood IL USA

**Keywords:** prostate, benign prostatic hyperplasia, BPH, fibrosis, antifibrotics, lower urinary tract dysfunction

## Abstract

Benign prostatic hyperplasia (BPH) is a widely prevalent age‐associated disease that is the main contributor to lower urinary tract dysfunction (LUTD) in aging men. Although prostate fibrosis has been recognized as a contributor to BPH pathophysiology, there are not any clinically available therapeutics that target this aspect of disease progression. In this study, we evaluated the antifibrotic potential of thalidomide using both *in vitro* and *in vivo* models of BPH/LUTD. Using benign human prostate stromal cells stimulated with transforming growth factor β‐1 (TGFβ1) followed by targeted transcriptomic profiling and assessment of canonical TGFβ signaling, we demonstrate that thalidomide attenuates expression of profibrotic genes, including extracellular matrix components. In aged male mice with LUTD, thalidomide administration led to a reduction in prostate collagen deposition and decreased organization of collagen fiber alignment. Functionally, thalidomide treatment improved LUTD in aged male mice, while prostate mass, androgen receptor expression and downstream signaling targets, and proliferative index remained unchanged, suggesting that the observed therapeutic effects are primarily mediated by antifibrotic mechanisms. Our findings highlight thalidomide's potential to modulate prostatic fibrosis and improve voiding function and support further investigation into the role of antifibrotic therapies as novel treatments for BPH/LUTD. © 2026 The Author(s). *The Journal of Pathology* published by John Wiley & Sons Ltd on behalf of The Pathological Society of Great Britain and Ireland.

## Introduction

Benign prostatic hyperplasia (BPH) arises from a loss of tissue homeostasis within the prostate and is the leading cause of lower urinary tract dysfunction (LUTD) in aging men [1, 2]. Histological BPH manifests clinically as lower urinary tract symptoms (LUTS), including urinary frequency, urgency, and nocturia. It is estimated that up to 80% of men over the age of 70 years will experience some degree of LUTS [3, 4]. Clinically, 5α‐reductase inhibitors (5ARIs) and α‐1 adrenergic receptor antagonists (α‐blockers, AB), are widely used for medical management as they alleviate symptoms and limit disease progression [5]. Despite the widespread use of AB and 5ARIs, up to 30% of patients ultimately fail to respond to medical management, necessitating invasive surgical interventions to alleviate their symptoms [6]. The failure of conventional therapies suggests that there may be alternative mechanisms that promote BPH/LUTS which AB and 5ARIs do not address. Men suffering from LUTS unresponsive to current medication approaches commonly have fibrotic prostates in which LUTS severity is out of proportion to the size of the gland, giving credence to the concept that prostatic fibrosis rather than bulk may explain these incongruencies.

While BPH has been attributed to prostate stromal and glandular overgrowth and urogenital smooth muscle dysfunction, recent work has implicated prostatic fibrosis as a key driver of LUTD [7, 8, 9, 10]. Aging, chronic inflammation, hormonal imbalance, and mitochondrial dysfunction promote extracellular matrix (ECM) remodeling, leading to fibrotic stiffening of the prostate and prostatic urethra, which may reduce urethral compliance and impair urinary outflow [8, 11, 12, 13, 14]. Notably, our group has demonstrated that fibrotic remodeling within the prostate transition zone (PTZ) correlates with an increased risk of BPH/LUTS progression, highlighting fibrosis as a promising therapeutic target [5, 15].

Thalidomide, a synthetically derived glutamic acid derivative initially developed as an antiemetic, has been shown to exhibit robust immunomodulatory and antifibrotic properties [16, 17, 18, 19]. Thalidomide has reemerged as a clinically valuable therapeutic agent that is approved by the US Food and Drug Administration (FDA) for diseases characterized by chronic inflammation and fibrosis, such as multiple myeloma, AIDS‐related aphthous stomatitis, and erythema nodosum leprosum [20]. Mechanistically, thalidomide exerts its multitude of effects by modulating cereblon (CRBN), an E3 ubiquitin ligase substrate receptor, leading to downstream degradation of transcription factors involved in canonical inflammatory and fibrotic pathways [21, 22]. Notably, thalidomide has been shown to suppress transforming growth factor β‐1 proprotein (TGFβ1) signaling, a central driver of fibrosis, by inhibiting SMAD2/3 activation and reducing ECM remodeling and collagen deposition in a variety of fibrotic disease states such as idiopathic pulmonary fibrosis, hepatic cirrhosis, cardiac, renal, and skin fibrosis [23, 24, 25, 26, 27]. In addition to its effects on canonical TGFβ signaling, thalidomide has been shown to decrease tumor necrosis factor α and other proinflammatory cytokines implicated in fibrotic remodeling [26, 27, 28, 29, 30]. Despite these well documented antifibrotic effects in other tissues and organ systems, the role of thalidomide in prostatic fibrosis mediated LUTD remains unexplored. Given the growing evidence linking fibrosis to BPH/LUTS progression, thalidomide posits itself a promising therapeutic for mitigating fibrotic remodeling and restoring functional tissue dynamics in the aging prostate. Furthermore, due to its preexisting FDA approval, thalidomide and its analogues are promising candidates for future clinical trials.

In this study, we assessed the potential of thalidomide to function as an antifibrotic agent in a preclinical mouse model of BPH/LUTS. Specifically, we utilized both *in vitro* and *in vivo* approaches to evaluate the impact of thalidomide on profibrotic signaling, collagen remodeling, and urinary function.

## Materials and methods

### Ethical approval

All animal experiments were conducted under the protocols approved by the University of Wisconsin Animal Care and Use Committee (registration number: M007019).

### Animals

Male C57Bl/6J mice (age 24 months; National Institute of Aging, Bethesda, MD, USA) were housed under standard laboratory conditions with 12:12 light/dark cycles and with food and water provided *ad libitum*. Mice were treated with (+/−)‐thalidomide (ThermoFisher Scientific, Waltham, MA, USA) at 10 mg/kg or vehicle (0.1% DMSO), 5 days per week, for 6 weeks via intraperitoneal injection (i.p.). Mice were euthanized with carbon dioxide followed by cardiac puncture. Three caliper measurements (*x*, *y*, *x*) were taken for each bladder, and volume was calculated by 4/3·*π*·((*x*·*y*·*z*)/8). Urogenital tracts (UGTs) were microdissected and weighted as previously described [10]. Tissues were fixed in 10% normal buffered formalin (NBF) before being paraffin embedded and sectioned at 5 μm.

### Void spot assays

Void spot assays (VSAs) were performed as previously described [31]. In brief, mice were placed on a 16 × 26‐cm Grade 238 chromatography paper (Ahlstrom, Kaukauna, WI, USA) for 4 h with access to food but not water. Filter papers were imaged using the UVP ChemStudio AnalytikJena (AnalytikJena, Jena, Germany) imager under ultraviolet light using an ethidium bromide filter and 500 ms exposure. Images were imported into ImageJ (National Institues of Health) and void spots analyzed using VoidWhizzard [32].

### Cell culture and reagents

Benign human prostate stromal cells (BHPrS1) [33] were grown in RPMI‐1640 (Corning, Durham, NC, USA) supplemented with 5% fetal bovine serum (Gibco, Grand Island, NY, USA), 0.2% normacin (InvivoGen, San Diego, CA, USA), and 1% HEPES buffer (Corning) at 37°C with 5% CO_2_. Recombinant human TGFβ1 (R&D Systems, Minneapolis, MN, USA) was solubilized and acid activated in 4 mm HCl containing 1 mg/ml BSA (Dot Scientific, Burton, MI, USA). Thalidomide was solubilized in cell culture‐grade DMSO. Cells were treated with vehicle (DMSO, 0.1%, HCL, 2 μm, BSA, 0.5 μg/ml) or TGFβ1 (10 ng/ml) ± thalidomide (100 μm).

### RNA isolation, first‐strand cDNA synthesis, and quantitative PCR

Cells were plated 1.0 × 10^5^ cells/well in a six‐well plate and allowed to adhere for 24 h in complete medium without serum starvation. After 24 h, cells were treated. After a 48 h treatment period, cells were harvested using 0.05% trypsin–EDTA (ThermoFisher Scientific). RNA was extracted using the RNeasy Mini Kit (Qiagen, Hilden, Germany). cDNA synthesis was performed using 1 μg RNA and the iScript cDNA synthesis (BioRad, Hercules, CA, USA). Gene expression was quantified using validated primers against *COL1A1*, *COL3A1*, and *COL5A1* (BioRad). Gene expression was normalized to *TBP* and *YWHAZ*. Experiments were performed in triplicate.

### NanoString

Total RNA (100 ng) was used with nCounter FibrosisV2 Panel kit (NanoString Technologies Inc., Seattle, WA, USA) to measure gene expression of 770 genes across 51 annotated pathways following the manufacturer's protocol as previously described [34]. Biological quadruplet samples were submitted for each treatment group: BHPrS1 + vehicle, BHPrS1 + TGFβ1, and BHPrS1 + TGFβ1 + thalidomide. Digital data were then transferred to nSolver 4.0 (NanoString Technologies Inc.) for quality control analysis and computing. Transcript read distributions and heat maps were generated as part of the QC step for each sample. Normalization to housekeeping genes, fold changes, and *p* values were calculated based on criteria provided by NanoString.

### Histology and immunohistochemistry

Formalin‐fixed and paraffin embedded tissue sections were stained with H&E. Immunohistochemistry was performed as previously described [10]. In brief, 5 μm tissue sections were cleared and rehydrated. Antigen retrieval was performed using a Decloaking Chamber (Biocare Medical, Pacheco, CA, USA) in 10 mm citrate buffer, pH 6.0, for 15 min at 110°C. Sections were incubated with anti‐Nkx3.1 (Proteintech, cat# 13069‐1‐AP; 1:1,000), anti‐Ki‐67 (Vector Laboratories, cat# VP‐K452; 1:1,000), and anti‐androgen receptor (Abcam, cat# ab227678; 1:1,000) at 4°C overnight. Horseradish peroxidase (HRP)‐conjugated horse anti‐rabbit IgG polymer reagent (Vector Laboratories, cat# MP‐7401) was used with 3,3′‐diaminobenzidine (DAB) chromogen (Cell Signaling Technology, cat# 8059) and hematoxylin nuclear counterstain.

### Immunocytochemistry

BHPrS1 cells were plated and treated as described above. Cells were washed and fixed using 4% paraformaldehyde. Cells were blocked with 1% normal horse serum and incubated at 4°C overnight with anti‐vimentin (Bioss, cat# BS‐0756R; 1:100). Cells were then incubated with donkey anti‐rabbit AlexaFluor 594 (ThermoFisher Scientific, cat# A32754), followed by 4′,6‐diamidino‐2‐phenylindole (DAPI) (ThermoFisher Scientific, D1306). Images were captured on the Leica DM4B (Leica Microsystems, Wetzlar, Germany), with three representative fields of view imaged per treatment. Quantification of the intracellular staining was quantified using ImageJ (NIH) to acquire the integrated density for the fluorophore channel (red) and to count cells. The integrated density of a field of view was normalized to the total cells count. Fields of view were averaged for each treatment and biological triplicates were performed.

### Western blotting

BHPrS1 cells were plated and treated as described above. Cells were lysed in radio‐immunoprecipitation assay (RIPA) buffer and protein was quantified using the detergent compatible (DC) protein assay (BioRad). Protein (15 μg) was resolved by SDS‐PAGE using a 4–20% gradient gel (BioRad) and transferred to a 0.2 μm PVDF membrane (BioRad). The membranes were probed with primary antibodies anti‐phosphorylated SMAD1/3/5 (phospho S423 + S425) (Abcam, cat# ab‐51451; 1:2,000), and anti‐SMAD3 (Cell Signaling Technology, cat# 9523S; 1:1,000) in 5% milk. Following, the membranes were probed with HRP‐labeled secondary (Fortis Life Sciences, Montgomery, TX, USA; cat#: A120‐201P; 1:10,000) and visualized using a ChemiDoc XRS+ chemiluminescence system (BioRad). Densitometric intensities of each sample using five biological replicates were performed.

### Picrosirius red staining and analysis

Prostatic urethra midpoint was identified from serial sections as previously described [35]. To analyze collagen bundle density, picrosirius red (PSR) staining was performed as previously described [36]. Sections were imaged at 20× under circular polarized light using the Nikon Eclipse E600 microscope and Nikon D2Fi2 camera (Nikon Instruments, Melville, NY, USA). Collagen birefringence was quantified using macros in ImageJ (NIH). Pixel counts were totaled across vehicle and thalidomide treated tissues and normalized to the sum of total pixels from the thresholded brightfield image.

### Curvelet transform‐fiber extraction (CT‐FIRE) analysis

Color corrected PSR images were converted into 8‐bit gray scale and batch analyzed in CT‐FIRE for collagen fiber length, straightness, angle, alignment, and orientation as previously described [37, 38].

### Tissue image analysis

Multispectral images were captured using Mantra (Perkin‐Elmer, Waltham, MA, USA) and quantified using Inform software (Perkin‐Elmer, Waltham, MA, USA) as previously described [39]. Expression of AR‐positive cells, Nkx3.1‐positive cells, Ki67‐positive cells were measured using a two‐bin positive/negative scoring system in InForm (https://www.inform-software.com/en/).

### Statistical analyses

qPCR and VSA were analyzed using an ordinary one‐way analysis of variance (ANOVA) with Tukey's correction for multiple comparisons. NanoString nCounter FibrosisV2 was analyzed using the nSolver 4.0 software (NanoString Technologies Inc.). PSR, CT‐FIRE, and IHC data were assessed for statistical significance using two‐way ANOVA with Tukey's honestly significant different (HSD) *post hoc* tests as well as Student's *t*‐test, when necessary. All analyses were performed using Graphpad Prism (La Jolla, CA, USA).

## Results

### Thalidomide attenuates TGFβ1‐induced profibrotic signaling and gene expression in BHPrS1 cells

**Figure 1:**
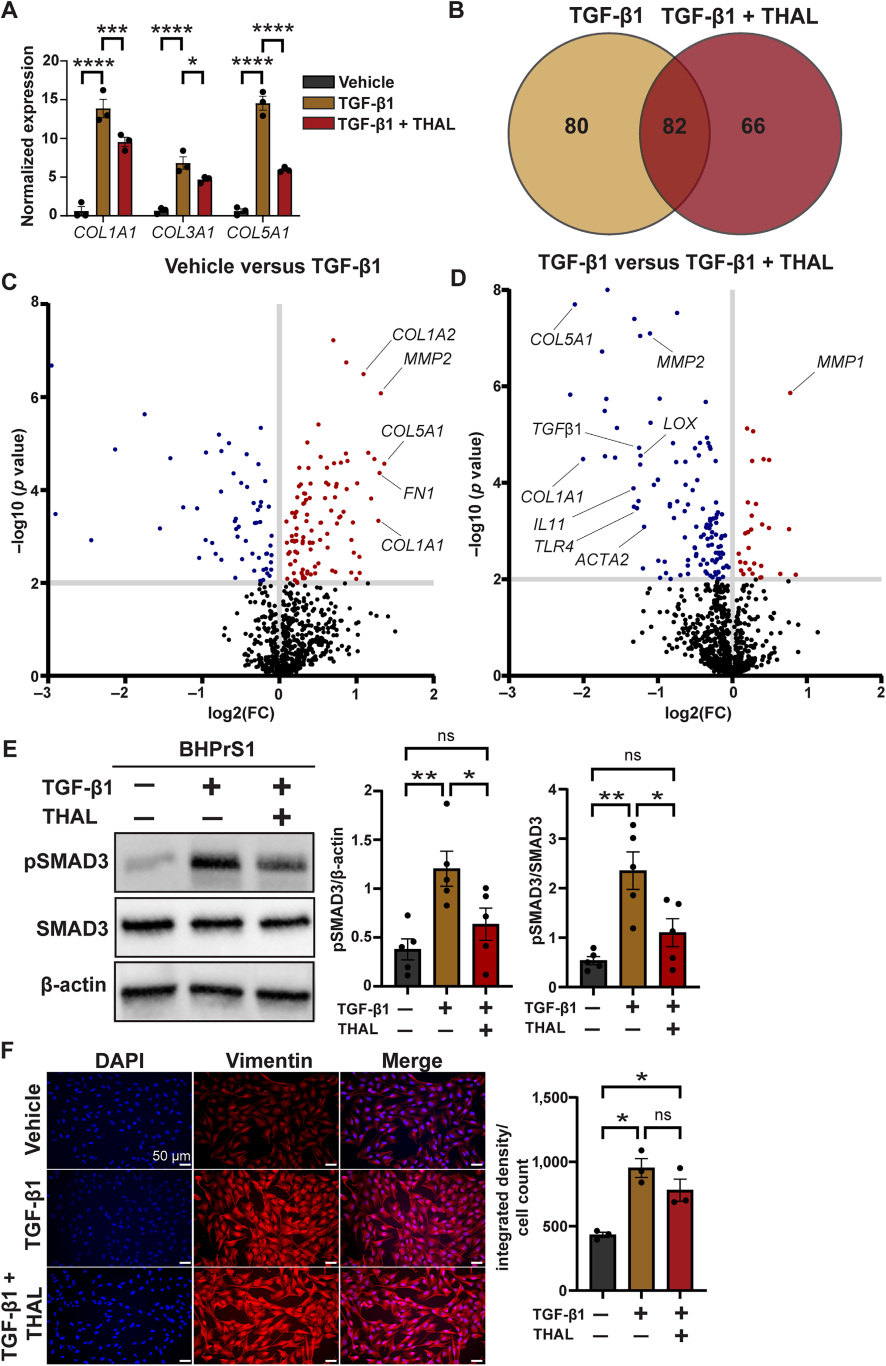
Thalidomide attenuates transforming growth factor β‐1 (TGFβ1)‐induced profibrotic signaling and gene expression in benign human prostate stromal (BHPrS1) cells. (A) BHPrS1 cells were treated with vehicle or TGFβ1 (10 ng/ml) ± thalidomide (100 μm) for 48 h. RNA was extracted and quantitative PCR performed for fibrosis‐related genes (*COL1A1*, *COL3A1*, *COL5A1*). (B) Venn diagram of differentially expressed genes between vehicle and TGFβ1 (yellow) and between TGFβ1 and TGFβ1 + thalidomide (THAL, red). (C, D) Volcano plots showing differentially expressed genes comparing (C) vehicle versus TGFβ1 and (D) TGFβ1 versus TGFβ1 + thalidomide. Red points indicate significantly upregulated transcripts, and blue points indicate significantly downregulated transcripts. Dashed line represents −log10(*p* value) = 2 threshold. (E) Western blotting of pSMAD3 and SMAD3 following a 15 min stimulation with vehicle or TGFβ1 (10 ng/ml) ± thalidomide (100 μm). (F) Representative ICC of vimentin expression following 48 h treatment with vehicle or TGFβ1 (10 ng/ml) ± thalidomide (100 μm). Vimentin (red) and nuclei (4′,6‐diamidino‐2‐phenylindole [DAPI], blue) were visualized by fluorescence microscopy (20×). Vimentin signal intensity (integrated density) was normalized to total cell count. Data are presented as mean ± SEM, *p < 0.05, **p < 0.01, ***p < 0.001, ****p < 0.0001.

Previous studies have shown that thalidomide may modulate the expression of proinflammatory and profibrotic genes [40, 41, 42]. To determine whether acute treatment of thalidomide has similar effects in benign human prostate cells, we stimulated BHPrS1 cells with TGFβ1 to induce a profibrotic phenotype and cotreated with thalidomide. As expected, qPCR analysis showed that TGFβ1 stimulation induced a robust increase in expression of profibrotic genes *COL1A1*, *COL3A1*, and *COL5A1*. Cotreatment with thalidomide attenuated this expression (Figure 1A). To assess for broader transcriptomic changes, we employed a targeted gene expression analysis using the NanoString nCounter Fibrosis V2 panel to assess expression of 770 fibrosis‐associated genes. Analysis showed 162 significantly altered genes in the vehicle versus TGFβ1 treatment group and 148 significantly altered genes in the TGFβ1 versus TGFβ1 + thalidomide treatment group, with 88 and 66 uniquely altered genes in either group, respectively (Figure 1B). TGFβ1 stimulation upregulated profibrotic genes such as *COL1A1*, *COL5A1*, *COL1A2* as well as ECM modulators *MMP2* (Figure 1C). Thalidomide cotreatment with TGFβ1 attenuated expression of many of the same genes while also downregulating proinflammatory genes such as *IL11* and canonical TGFβ pathway modulators such as *TGFβ1* (Figure 1D). A list of the top 25 significantly upregulated and downregulated genes in the vehicle versus TGFβ1 group, and TGFβ1 versus TGFβ1 + thalidomide group can be found in the supplementary material, Tables S1 and S2, respectively.

Consistent with transcriptional findings, western blotting demonstrated a significant reduction in TGFβ‐induced phosphorylation of SMAD3 and pSMAD3/SMAD3 ratio with thalidomide cotreatment supporting inhibition of canonical TGFβ signaling (Figure 1E). However, immunocytochemistry (ICC) did not show a significant attenuation of TGFβ1‐induced expression of intracellular vimentin, a marker of myofibroblast differentiation, in thalidomide cotreated cells (Figure 1F).

### Thalidomide attenuates fibrosis in the mouse lower urogenital tract

**Figure 2:**
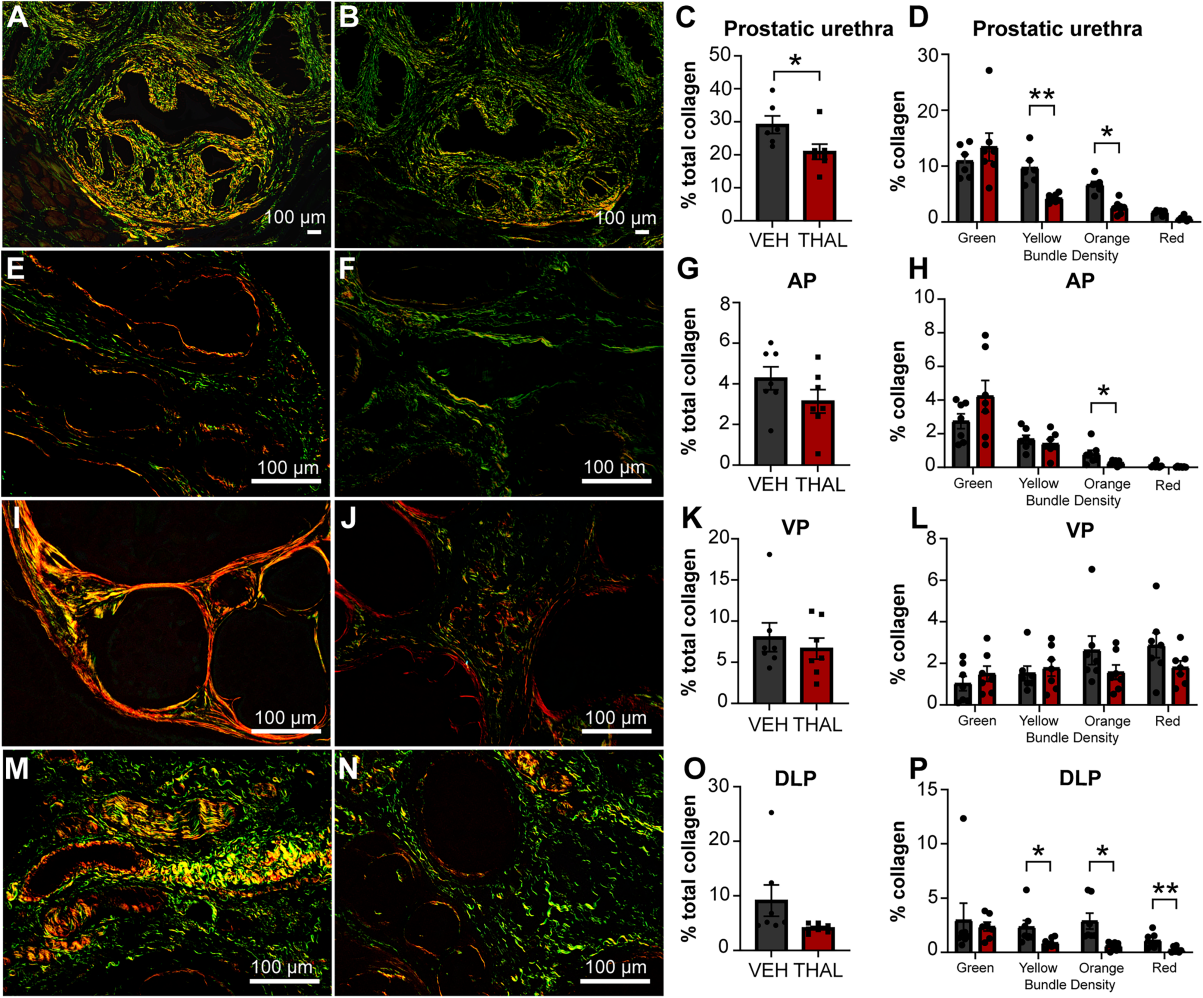
Thalidomide attenuates fibrosis in the mouse prostatic urethra and prostate. (A, B) Representative picrosirius red (PSR)‐stained sections of the prostatic urethra from (A) vehicle‐treated and (B) thalidomide‐treated mice. (C, D) Quantification of (C) total collagen area (as a percentage of total tissue) and (D) birefringence‐dependent collagen bundle density (green, yellow, orange, red) in the prostatic urethra. (E, F) Representative PSR‐stained sections of the anterior prostate (AP) from (E) vehicle‐treated and (F) thalidomide‐treated mice. (G, H) Quantification of (G) total collagen area and (H) collagen bundle density in AP. (I, J) Representative PSR stained sections of the ventral prostate (VP) from (I) vehicle‐treated and (J) thalidomide‐treated mice. (K, L) Quantification of (K) total collagen and (L) collagen bundle density in VP. (M, N) Representative PSR‐stained sections of the dorsolateral prostate (DLP) from (M) vehicle‐treated and (N) thalidomide‐treated mice. (O, P) Quantification of (O) total collagen and (P) collagen bundle density in DLP. Mice received vehicle (VEH) or thalidomide (THAL) (10 mg/kg, i.p.), 5 days per week for 6 weeks. Collagen birefringence was quantified using circular polarized light. Data are presented as mean ± SEM, *p < 0.05, **p < 0.01.

We have previously shown that aged mice show evidence of prostate and periurethral fibrosis, like that of patients with BPH [8, 15]. To determine if thalidomide treatment would have similar antifibrotic effects *in vivo*, we treated aged C57BL/6J mice with thalidomide five times per week for 6 weeks. Following euthanasia, UGTs were harvested and assessed for fibrosis using birefringent PSR staining and circular polarized microscopy. Representative images are shown in Figure 2, where panels A, E, I, and M depict vehicle‐treated tissues from the prostatic urethra, anterior prostate (AP), ventral prostate (VP) and dorsolateral prostate (DLP), respectively, and panels B, F, J, and N show the corresponding thalidomide‐treated tissues. Mice treated with thalidomide had a significant decrease in total collagen content within the prostatic urethra region (Figure 2C). Furthermore, we observed significant decreases in thick collagen bundles; yellow and orange (Figure 2D). We then assessed fibrosis within the individual prostate lobes. While there was no significant difference in total collagen content within the AP (Figure 2G), VP (Figure 2K), or DLP (Figure 2O), analysis of the individual bundle densities revealed lobe‐specific differences. In the AP, thalidomide significantly reduced the density of thick orange collagen bundles (Figure 2H), whereas in the DLP, yellow, orange, and red bundles were significantly decreased (Figure 2P). No significant differences in bundle density were observed in the VP (Figure 2L). In addition to PSR, overall tissue morphology was assessed using H&E staining. We did not observe any gross differences in inflammation or immune cell infiltration between groups. However, imaging revealed visually thicker periglandular stroma, particularly surrounding the prostatic ducts in the AP and DLP in vehicle‐treated mice. A similar pattern was observed in the prostatic urethra region of vehicle‐treated mice (supplementary material, Figure S1).

### Thalidomide modulates individual collagen fiber characteristics in the mouse prostatic urethra

ECM organization, specifically collagen network superstructure, has been shown to be associated with pathology and disease progression in a variety of diseases including idiopathic pulmonary fibrosis, ovarian cancer, and pancreatic cancer. We performed CT‐FIRE analysis on the color corrected birefringent PSR images from the prostate lobes and prostatic urethra. Analysis showed that collagen fiber alignment was significantly decreased in the prostatic urethra region of thalidomide treated mice compared with controls. Moreover, we observed a significant reduction in collagen fiber angle within the DLP of thalidomide‐treated mice. Complete collagen fiber metrics are summarized in Table 1.

**Table 1:** Collagen metrics in vehicle versus thalidomide treated mice.

Fiber	Mean ± SEM	
	Vehicle	Thalidomide
Anterior prostate		
Length	55.34 ± 1.23	55.02 ± 1.59
Straightness	0.92 ± 0.002	0.93 ± 0.001
Angle	110.4 ± 3.08	116.9 ± 2.34
Alignment	0.13 ± 0.03	0.14 ± 0.02
Orientation	150 ± 4.47	139.6 ± 3.55
Ventral prostate		
Length	58.85 ± 2.73	54.97 ± 1.92
Straightness	0.93 ± 0.001	0.94 ± 0.001
Angle	114.3 ± 2.31	110 ± 5.11
Alignment	0.11 ± 0.01	0.11 ± 0.03
Orientation	132.3 ± 7.56	109.9 ± 17.00
Dorsolateral prostate		
Length	54.41 ± 0.76	53.05 ± 1.65
Straightness	0.92 ± 0.001	0.93 ± 0.001
Angle	114.6 ± 2.54*	106.7 ± 2.37*
Alignment	0.18 ± 0.02	0.14 ± 0.01
Orientation	143.3 ± 1.80	142.8 ± 4.21
Prostatic urethra		
Length	58.26 ± 0.97	57.25 ± 0.97
Straightness	0.92 ± 0.001	0.93 ± 0.004
Angle	99.55 ± 1.74	99.19 ± 1.36
Alignment	0.24 ± 0.009*	0.17 ± 0.01*
Orientation	140.8 ± 6.89	140.9 ± 4.24

* Vehicle versus thalidomide unpaired Student's *t*‐test *p* < 0.05.

### Thalidomide ameliorates LUTD in aged mice

**Figure 3:**
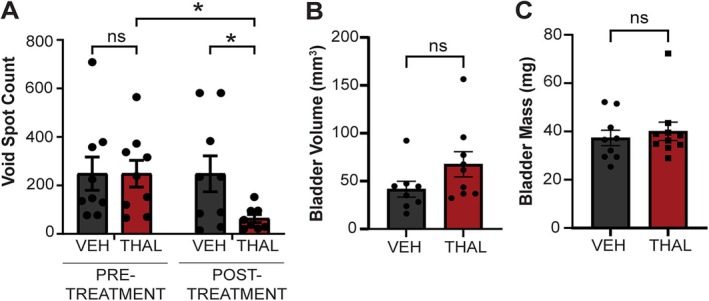
Thalidomide treatment attenuates lower urinary tract dysfunction in aged mice. (A) Void post assay showing total spot count in vehicle‐treated and thalidomide‐treated mice pre‐ and post‐treatment. (B, C) Post‐mortem bladder morphometrics including (B) bladder volume and (C) bladder mass in vehicle‐treated and thalidomide‐treated mice. Mice received vehicle (VEH) or thalidomide (THAL) (10 mg/kg, i.p.), 5 days per week for 6 weeks. Voiding behavior was assessed by void spot assay and bladders were harvested for volumetric and weight analysis. Data are presented as mean ± SEM, *p < 0.05.

We have previously shown that aged C57BL/6J mice spontaneously develop LUTD that closely phenocopies that of aging men with BPH/LUTS [8]. To evaluate the effectiveness of thalidomide as a potential therapy to alleviate LUTD, we assessed for urinary frequency at baseline and at the end of the study. Mice treated with thalidomide had a significant improvement in LUTD as evident by a decrease in urinary frequency on VSA compared with vehicle‐treated mice (Figure 3A). However, bladder volume (Figure 3B) and bladder mass (Figure 3C) remained unchanged.

### Thalidomide does not function as an antiandrogen or antiproliferative agent to improve urinary function

**Figure 4:**
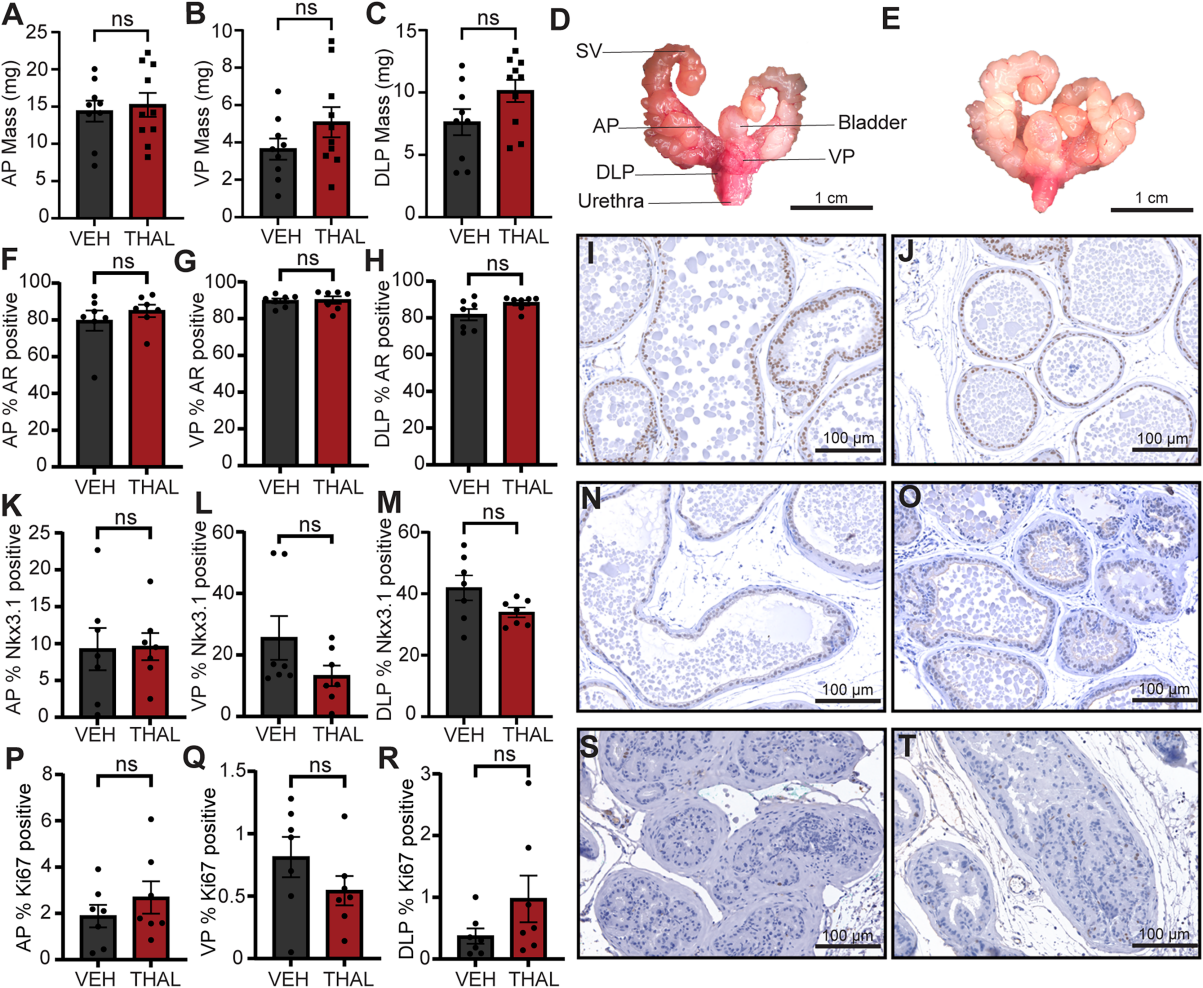
Thalidomide does not function as an antiandrogen or antiproliferative agent to improve urinary function. (A–C) Post‐mortem prostate lobe masses in vehicle‐ and thalidomide‐treated mice: (A) anterior prostate (AP), (B) ventral prostate (VP), and (C) dorsolateral prostate (DLP). (D, E) Representative *ex vivo* images of *en bloc* mouse urogenital tracts from (D) vehicle‐ and (E) thalidomide‐treated mice. (F–H) Quantification of androgen receptor (AR)‐positive nuclei (DAB, brown) in (F) AP, (G) VP, and (H) DLP. (I, J) Representative AR IHC images from DLP of (I) vehicle‐ and (J) thalidomide‐treated mice (similar trends were observed in AP and VP). (K–M) Quantification of Nkx3.1‐positive nuclei (brown) in (K) AP, (L) VP, and (M) DLP. (N, O) Representative Nkx3.1 IHC images from DLP of (N) vehicle‐ and (O) thalidomide‐treated mice (similar trends were observed in AP and VP). (P–R) Quantification of Ki67‐positive nuclei (brown) in (P) AP, (Q) VP, and (R) DLP. (S, T) Representative Ki67 IHC images from DLP of (S) vehicle‐ and (T) thalidomide‐treated mice (similar trends were observed in AP and VP). Mice received vehicle (VEH) or thalidomide (THAL) (10 mg/kg, i.p.), 5 days per week for 6 weeks. Tissue sections were stained for AR, Nkx3.1, and Ki67, and positive nuclei were quantified. AP, anterior prostate; VP, ventral prostate; DLP, dorsolateral prostate; SV, seminal vesicle. Data are presented as mean ± SEM.

There have been reports of thalidomide having antiandrogenic effects, specifically in that thalidomide has been shown to reduce prostate‐specific antigen (PSA) levels in a subset of men with androgen‐independent prostate adenocarcinoma [43, 44, 45, 46]. To determine whether the improvement in LUTD involves antiandrogenic or antiproliferative effects, prostate lobes were evaluated for mass, androgen receptor (AR) signaling, and proliferative index. We did not observe any differences in AP, VP, or DLP mass between treatment groups (Figure 4A–C) with no gross overall difference in prostate morphology (Figure 4D, E). IHC revealed no lobe‐specific differences between treatment groups in AR expression (Figure 4F–J), Nkx3.1 expression (Figure 4K–O), or Ki‐67 positivity (Figure 4P–T).

## Discussion

BPH is an age‐related condition that results in the loss of prostate tissue homeostasis and clinically manifests as LUTD. Although prostatic proliferation and urogenital smooth muscle dysfunction have classically been implicated in BPH, emerging data suggest that prostatic fibrosis plays a critical role in disease progression and resistance to medical therapies [7, 8, 9, 15, 47]. In this study, we evaluated the effectiveness of thalidomide as an antifibrotic agent and its ability to improve LUTD in an aged mouse model of BPH/LUTS. Our findings demonstrate that thalidomide attenuates profibrotic signaling pathways and ECM remodeling in both *in vitro* and *in vivo* models. Collectively, these data support the role for thalidomide and its analogues as a potential antifibrotic agent for the treatment of BPH and associated LUTD.

Aberrant tissue fibrosis is a process that involves unregulated inflammation, fibroblast activation, proliferation, and myofibroblast conversion, and subsequent deposition of ECM. Targeted transcriptome‐wide profiling showed that thalidomide modulates multiple canonical pathways implicated in fibrosis. Cotreatment of TGFβ1‐stimulated BHPrS1 cells with thalidomide attenuated the expression of a broad set of TGFβ1‐induced genes involved in ECM synthesis, matrix remodeling, and proinflammatory signaling. Notably, thalidomide treatment downregulated expression of fibrillar collagen subunits *COL1A1*, *COL1A2*, *COL5A1*, and fibronectin (*FN1*). Furthermore, *SERPINE1*, an inhibitor of the plasmin‐MMP system and known to be upregulated in fibrotic liver, lung, and kidney diseases, was also downregulated [48]. A marker of myofibroblast trans‐differentiation, *ACTA2*, was also among those that were downregulated. Our protein‐level analysis is consistent with previous reports demonstrating that thalidomide inhibits canonical TGFβ signaling [29, 42]. However, the absence of a corresponding decrease in vimentin expression by ICC suggests that thalidomide may not fully suppress myofibroblast activation. Alternatively, it is possible that vimentin expression remained elevated through non‐canonical TGFβ signaling pathways (e.g. PI3K/ATK, ERK, MAPK) which promote cytoskeletal changes independent of SMAD activation.

Chronic inflammation is a nidus for fibrosis. Transcriptomic analysis showed that thalidomide attenuates expression of proinflammatory mediators such as *IL6*, *IL11*, and *TLR4*, all of which have been implicated in tissue fibrosis [49, 50, 51]. While we did not find any gross inflammatory differences observed by H&E, subtle immunomodulatory effects of thalidomide on the prostate cannot be excluded without a more robust analysis. Most interestingly, however, was the finding that thalidomide also modulates expression of ECM modulators such as *MMP2* (gelatinase‐A), *MMP13* (collagenase‐3), and *MMP1* (interstitial collagenase). Maintenance of the ECM is a balance between production of components including collagens, fibronectin, and elastin and degradation of components by matrix metalloproteinases [48]. In the context of hepatic fibrosis, loss of *MMP13* attenuated fibrotic remodeling through decreased proinflammatory cytokine signaling, ECM production, and myofibroblast activation [52]. Taken together, these findings suggest that thalidomide may not only inhibit the production and deposition of the ECM, but it may also change the structure of existing collagen fiber networks.

These transcriptional changes were reflected in our *in vivo* studies. While total collagen content did not differ in the different mouse prostate lobes, analysis of individual collagen bundles revealed significant reductions in thick collagen fibers particularity in the AP and DLP. In the mouse prostatic urethra, a region where we and others have observed to have the most anatomical homology with the human PTZ, thalidomide reduced total collagen content and the proportion of thick collagen bundles. These fiber populations (yellow, orange, and red) are mature, established, densely organized collagen fibers and have been associated with increased matrix stiffness [53, 54]. Intermolecular cross‐linking of collagen provides tensile strength to tissues, affects trafficking of immune cells, and affects tissue function [55]. CT‐FIRE analysis revealed that thalidomide decreased alignment and angle of individual collagen fibers in the prostatic urethra and DLP, respectively. We have previously shown that periurethral collagen fiber linearity and organization is associated with clinical progression in patients with BPH; specifically, patients whose condition progressed had a higher degree of fiber alignment and angle [15]. The DLP contributes a substantial proportion of ducts to the prostatic urethra region, suggesting that the changes observed in the DLP likely also manifest in the periurethral collagen network [35]. This reduction in a highly aligned periurethral collagen network suggests changes to the tissue microenvironment leading to a less rigid, more compliant prostatic urethra, alleviating LUTD. Supported by the structural changes at the tissue level, we hypothesize that the reduction in collagen alignment may be due in part by thalidomide‐mediated downregulation of the enzyme lysyl oxidase, which generates collagen cross linkages. ECM with decreased collagen cross linkages are susceptible to metalloproteinase‐mediated degradation in aged skin, which is a potential mechanism herein, given *MMP1* upregulation [56, 57]. However, direct mechanistic studies are needed to clarify the role of thalidomide in ECM remodeling in the prostate.

Preclinical and clinical studies have reported that thalidomide may exhibit antiandrogen properties. Due to structural similarity with the nonsteroidal antiandrogen *N*(3,5‐dimethyl‐4‐isoxazolymethyl)phthalimide (DIMP), it has been hypothesized that thalidomide may bind to and antagonize the androgen receptor [58, 59]. As such, gynecomastia and sexual dysfunction have been reported with the administration of high‐dose thalidomide [60]. Several phase II clinical studies in patients with advanced androgen‐independent prostate cancer have observed a 50% reduction in circulating PSA in 18–53% of enrolled patients [43, 44, 45, 46]. However, in our study, we did not observe evidence supporting the finding that thalidomide may function as an antiandrogen or antiproliferative agent in the context of the aged mouse prostate. Prostate lobe masses remained unchanged following treatment, and we did not observe any significant changes in epithelial AR expression, Nkx3.1 positivity, or Ki67 positivity. Functionally, aged mice treated with thalidomide demonstrated improved voiding behavior as evidenced by decreased urinary frequency on VSA. Therefore, we hypothesize that the improvement in LUTD is primarily due to antifibrotic actions in the mouse lower UGT. Several reasons may account for the absence of antiandrogenic effects. We utilized lower doses and shorter treatment durations than the clinical trials and changes to AR signaling may require longer exposure. Moreover, the mouse AR is 83% homologous to the human AR, therefore differences in ligand binding domain or cofactor recruitment may reduce susceptibility to thalidomide antagonism [61].

Our findings support the role of thalidomide in attenuating prostate and periurethral fibrosis and improving LUTD. However, limitations of this work exist. First, our study utilized an acute dosing model, and urinary function was assessed for 6 weeks. As AB and 5ARIs are taken chronically in the clinic, further analysis of the long‐term effects of thalidomide on urinary function should be assessed. Additionally, while our targeted transcriptomic analysis provided insight into the mechanism of action underlying thalidomide's antifibrotic effect, specific cellular targets should be determined. Future studies can leverage lineage marker tracing or single cell transcriptomic analysis to determine the stromal, epithelial, and immune population affected. Moreover, although we demonstrate the therapeutic potential of thalidomide, its clinical use is limited by known side effects, including coagulopathies, peripheral edema, gastrointestinal dysfunction, and peripheral neuropathies. Current FDA‐approved antifibrotics including pirfenidone and nintedanib are often poorly tolerated and require dose adjustment or polypharmacy to manage adverse effects, highlighting the need for better agents [62]. Derivatives of thalidomide, lenalidomide, and pomalidomide have been shown to be more effective while greatly reducing adverse effects [20, 63, 64]. Therefore, these findings may be viewed as proof of concept that modulation of tissue fibrosis is a valid treatment strategy in BPH/LUTS. Future preclinical studies should focus on these clinically relevant derivates and evaluate their antifibrotic capacity in the context of benign prostatic disease. Finally, while we focused on evaluating the antifibrotic and antiandrogenic effects of thalidomide, other studies have found that thalidomide (and its derivatives) may also function as a modulator of prostatic smooth muscle dysfunction [65, 66]. Interestingly, our weekly VSAs suggest that the improvement in voiding function with thalidomide treatment may occur relatively quickly following initiation (supplementary material, Figure S2). Although not significant, we saw a reduction in voiding as early as 1 week after starting therapy. This finding may suggest that thalidomide may exert more immediate effects on the lower UGT, possibly on smooth muscle like that of AB therapy, in contrast to 5ARI therapy which often takes months to achieve clinical effectiveness. These data support the possibility of thalidomide and its derivates functioning as a multimodal therapeutic, one that targets prostate fibrosis and smooth muscle dysfunction, two key components of BPH/LUTS pathophysiology and its medically nonresponsive phenotypes.

Here we demonstrate that thalidomide attenuates prostate and periurethral fibrosis by modulating profibrotic signaling pathways, ECM turnover, and collagen maturation, ultimately leading to improved LUTD in an aged mouse model of BPH/LUTD. These data suggest that targeting the extracellular tissue environment may alleviate prostate fibrosis mediated LUTS. Although additional studies are needed to define long‐term effects, specific cellular targets, and the translational potential of clinically relevant thalidomide derivates, our work supports the idea of further exploring antifibrotic agents as novel therapeutics for BPH‐related voiding dysfunction.

## Author contributions statement

ARL and WAR designed the overall study and acquired funding. ARL performed all experimentation. ARL created all figures with assistance from SMV and EAR. ARL analyzed the data and wrote the first draft of the manuscript. All subsequent drafts were reviewed and edited by ARL, SMV, EAR, MSL, KTM, ZF and WAR. All authors have read and agreed to the published version of the manuscript.

## Supporting information


**Figure S1.** Thalidomide attenuates lower urinary tract dysfunction in aged mice
**Figure S2**. Thalidomide does not lead to gross differences in inflammatory infiltrate in the mouse urogenital tract
**Table S1**. Top differentially expressed genes in benign human prostate stromal cells treated with transforming growth factor β‐1 (TGFβ1) compared with vehicle
**Table S2**. Top differentially expressed genes in benign human prostate stromal cells treated with transforming growth factor β‐1 (TGFβ1) + thalidomide compared with TGFβ1
**Table S3**. Forward and reverse primer sequences for housekeeping genes
**Table S4**. Selected target amplicon context sequences for BioRad validated primers

## Data Availability

The data that support the findings of this study are available from the corresponding author upon reasonable request.

## References

[1] Berry SJ , Coffey DS , Walsh PC , *et al*. The development of human benign prostatic hyperplasia with age. J Urol 1984; 132 **:** 474–479. 6206240 10.1016/s0022-5347(17)49698-4

[2] Kirby RS . The natural history of benign prostatic hyperplasia: what have we learned in the last decade? Urology 2000; 56 **:** 3–6. 11074195 10.1016/s0090-4295(00)00747-0

[3] Garraway WM , Collins GN , Lee RJ . High prevalence of benign prostatic hypertrophy in the community. Lancet 1991; 338 **:** 469–471. 1714529 10.1016/0140-6736(91)90543-x

[4] Parsons JK , Bergstrom J , Silberstein J , *et al*. Prevalence and characteristics of lower urinary tract symptoms in men aged ≥80 years. Urology 2008; 72 **:** 318–321. 18554695 10.1016/j.urology.2008.03.057 PMC2597492

[5] McConnell JD , Roehrborn CG , Bautista OM , *et al*. The long‐term effect of doxazosin, finasteride, and combination therapy on the clinical progression of benign prostatic hyperplasia. N Engl J Med 2003; 349 **:** 2387–2398. 14681504 10.1056/NEJMoa030656

[6] Kaplan SA . Factors in predicting failure with medical therapy for BPH. Rev Urol 2005; 7 **:** S34‐9. PMC1477626 16986060

[7] Bauman TM , Nicholson TM , Abler LL , *et al*. Characterization of fibrillar collagens and extracellular matrix of glandular benign prostatic hyperplasia nodules. PLoS One 2014; 9 **:** e109102. 25275645 10.1371/journal.pone.0109102 PMC4183548

[8] Liu TT , Thomas S , McLean DT , *et al*. Prostate enlargement and altered urinary function are part of the aging process. Aging (Albany NY) 2019; 11 **:** 2653–2669. 31085797 10.18632/aging.101938 PMC6535061

[9] Ma J , Gharaee‐Kermani M , Kunju L , *et al*. Prostatic fibrosis is associated with lower urinary tract symptoms. J Urol 2012; 188 **:** 1375–1381. 22906651 10.1016/j.juro.2012.06.007 PMC3485634

[10] Nicholson TM , Ricke EA , Marker PC , *et al*. Testosterone and 17β‐estradiol induce glandular prostatic growth, bladder outlet obstruction, and voiding dysfunction in male mice. Endocrinology 2012; 153 **:** 5556–5565. 22948219 10.1210/en.2012-1522 PMC3473198

[11] Adrian AE , Liu TT , Pascal LE , *et al*. Aging‐related mitochondrial dysfunction is associated with fibrosis in benign prostatic hyperplasia. J Gerontol A Biol Sci Med Sci 2024; 79 **:** glad222. 37738211 10.1093/gerona/glad222 PMC11083627

[12] He Q , Xu C , Guo J , *et al*. Bisphenol A exposure stimulates prostatic fibrosis via exosome‐triggered epithelium changes. Food Chem Toxicol 2024; 185 **:** 114450. 38215961 10.1016/j.fct.2024.114450

[13] Jin BR , Lim CY , Kim HJ , *et al*. Antioxidant mitoquinone suppresses benign prostatic hyperplasia by regulating the AR‐NLRP3 pathway. Redox Biol 2023; 65 **:** 102816. 37454529 10.1016/j.redox.2023.102816 PMC10368918

[14] Nicholson TM , Moses MA , Uchtmann KS , *et al*. Estrogen receptor‐α is a key mediator and therapeutic target for bladder complications of benign prostatic hyperplasia. J Urol 2015; 193 **:** 722–729. 25167991 10.1016/j.juro.2014.08.093 PMC4305478

[15] Macoska JA , Uchtmann KS , Leverson GE , *et al*. Prostate transition zone fibrosis is associated with clinical progression in the MTOPS study. J Urol 2019; 202 **:** 1240–1247. 31188728 10.1097/JU.0000000000000385 PMC7339116

[16] Santana AC , Andraus W , Silva FMO , *et al*. Immunomodulatory effects of thalidomide in an experimental brain death liver donor model. Sci Rep 2021; 11 **:** 19221. 34584130 10.1038/s41598-021-98538-z PMC8479052

[17] Sana Vilela V , Andrighetti De Oliveira Braga K , Moreira Ruiz L , *et al*. Anti‐inflammatory effect of thalidomide in an experimental lung donor model of brain death. Sci Rep 2024; 14 **:** 8796. 38627574 10.1038/s41598-024-59267-1 PMC11021429

[18] D'Amato RJ , Loughnan m , Flynn E , *et al*. Thalidomide is an inhibitor of angiogenesis. Proc Natl Acad Sci U S A 1994; 91 **:** 4082–4085. 7513432 10.1073/pnas.91.9.4082 PMC43727

[19] Holstein SA , McCarthy PL . Immunomodulatory drugs in multiple myeloma: mechanisms of action and clinical experience. Drugs 2017; 77 **:** 505–520. 28205024 10.1007/s40265-017-0689-1 PMC5705939

[20] Ghobrial IM , Rajkumar SV . Management of thalidomide toxicity. J Support Oncol 2003; 1 **:** 194–205. 15334875 PMC3134146

[21] Ito T , Handa H . Molecular mechanisms of thalidomide and its derivatives. Proc Jpn Acad Ser B Phys Biol Sci 2020; 96 **:** 189–203. 10.2183/pjab.96.016 PMC7298168 32522938

[22] Ito T , Ando H , Suzuki T , *et al*. Identification of a primary target of thalidomide teratogenicity. Science 2010; 327 **:** 1345–1350. 20223979 10.1126/science.1177319

[23] Chong L‐W , Hsu Y‐C , Chiu Y‐T , *et al*. Anti‐fibrotic effects of thalidomide on hepatic stellate cells and dimethylnitrosamine‐intoxicated rats. J Biomed Sci 2006; 13 **:** 403–418. 16604421 10.1007/s11373-006-9079-5

[24] Hosseini‐Chegeni A , Jazaeri F , Yousefi‐Ahmadipour A , *et al*. Thalidomide attenuates the hyporesponsiveness of isolated atria to chronotropic stimulation in BDL rats: the involvement of TNF‐α, IL‐6 inhibition, and SOCS1 activation. Iran J Basic Med Sci 2019; 22 **:** 1259–1266. 32128089 10.22038/ijbms.2019.32256.7742 PMC7038422

[25] Kang Y , Zhang C , He Y , *et al*. Thalidomide attenuates skin lesions and inflammation in rosacea‐like mice induced by long‐term exposure of LL‐37. Drug Des Devel Ther 2022; 16 **:** 4127–4138. 10.2147/DDDT.S393122 PMC9724583 36483458

[26] Tabata C , Tabata R , Kadokawa Y , *et al*. Thalidomide prevents bleomycin‐induced pulmonary fibrosis in mice. J Immunol 2007; 179 **:** 708–714. 17579094 10.4049/jimmunol.179.1.708

[27] Zhang H , Yang Y , Wang Y , *et al*. Renal‐protective effect of thalidomide in streptozotocin‐induced diabetic rats through anti‐inflammatory pathway. Drug Des Devel Ther 2018; 12 **:** 89–98. 10.2147/DDDT.S149298 PMC5765978 29386886

[28] Choe J‐Y , Jung H‐J , Park K‐Y , *et al*. Anti‐fibrotic effect of thalidomide through inhibiting TGF‐β‐induced ERK1/2 pathways in bleomycin‐induced lung fibrosis in mice. Inflamm Res 2010; 59 **:** 177–188. 19757088 10.1007/s00011-009-0084-9

[29] Liang CJ , Yen YH , Hung LY , *et al*. Thalidomide inhibits fibronectin production in TGF‐beta1‐treated normal and keloid fibroblasts via inhibition of the p38/Smad3 pathway. Biochem Pharmacol 2013; 85 **:** 1594–1602. 23500539 10.1016/j.bcp.2013.02.038

[30] Yeh TS , Ho YP , Huang SF , *et al*. Thalidomide salvages lethal hepatic necroinflammation and accelerates recovery from cirrhosis in rats. J Hepatol 2004; 41 **:** 606–612. 15464241 10.1016/j.jhep.2004.06.019

[31] Keil KP , Abler LL , Altmann HM , *et al*. Influence of animal husbandry practices on void spot assay outcomes in C57BL/6J male mice. NeurourolUrodyn 2016; 35 **:** 192–198. 10.1002/nau.22692 PMC4428995 25394276

[32] Wegner KA , Abler LL , Oakes SR , *et al*. Void spot assay procedural optimization and software for rapid and objective quantification of rodent voiding function, including overlapping urine spots. Am J Physiol Renal Physiol 2018; 315 **:** F1067–F1080. 29972322 10.1152/ajprenal.00245.2018 PMC6230749

[33] Franco OE , Jiang M , Strand DW , *et al*. Altered TGF‐β signaling in a subpopulation of human stromal cells promotes prostatic carcinogenesis. Cancer Res 2011; 71 **:** 1272–1281. 21303979 10.1158/0008-5472.CAN-10-3142 PMC3076790

[34] Magee K , Marsh IR , Turek mM , *et al*. Safety and feasibility of an in situ vaccination and immunomodulatory targeted radionuclide combination immuno‐radiotherapy approach in a comparative (companion dog) setting. PLoS One 2021; 16 **:** e0255798. 34383787 10.1371/journal.pone.0255798 PMC8360580

[35] Limkar AR , Sharma SM , Ricke WA . High‐resolution ex vivo nanoCT reveals 3D architecture of the adult male mouse lower urogenital tract. PLoS One 2025; 20 **:** e0326004. 40966215 10.1371/journal.pone.0326004 PMC12445489

[36] Whittaker P , Kloner RA , Boughner DR , *et al*. Quantitative assessment of myocardial collagen with picrosirius red staining and circularly polarized light. Basic Res Cardiol 1994; 89 **:** 397–410. 7535519 10.1007/BF00788278

[37] Bredfeldt JS , Liu Y , Pehlke CA , *et al*. Computational segmentation of collagen fibers from second‐harmonic generation images of breast cancer. J Biomed Opt 2014; 19 **:** 16007. 24407500 10.1117/1.JBO.19.1.016007 PMC3886580

[38] Liu Y , Keikhosravi A , Mehta GS , *et al*. Methods for quantifying fibrillar collagen alignment. Methods Mol Biol 2017; 1627 **:** 429–451. 28836218 10.1007/978-1-4939-7113-8_28 PMC6343484

[39] Zhang H , Liu TT , Ricke EA , *et al*. Prostatic androgen receptor signaling shows an age‐related and lobe‐specific alteration in mice. Sci Rep 2024; 14 **:** 30302. 39638850 10.1038/s41598-024-79879-x PMC11621416

[40] Choe JY , Jung HJ , Park KY , *et al*. Anti‐fibrotic effect of thalidomide through inhibiting TGF‐beta‐induced ERK1/2 pathways in bleomycin‐induced lung fibrosis in mice. Inflamm Res 2010; 59 **:** 177–188. 19757088 10.1007/s00011-009-0084-9

[41] Mazzoccoli L , Cadoso SH , Amarante GW , *et al*. Novel thalidomide analogues from diamines inhibit pro‐inflammatory cytokine production and CD80 expression while enhancing IL‐10. Biomed Pharmacother 2012; 66 **:** 323–329. 22770990 10.1016/j.biopha.2012.05.001

[42] Zhou XL , Xu P , Chen HH , *et al*. Thalidomide inhibits TGF‐β1‐induced epithelial to mesenchymal transition in alveolar epithelial cells via Smad‐dependent and Smad‐independent signaling pathways. Sci Rep 2017; 7 **:** 14727. 29116196 10.1038/s41598-017-15239-2 PMC5677010

[43] Drake MJ , Robson W , Mehta P , *et al*. An open‐label phase II study of low‐dose thalidomide in androgen‐independent prostate cancer. Br J Cancer 2003; 88 **:** 822–827. 12644816 10.1038/sj.bjc.6600817 PMC2377091

[44] Figg WD , Arlen P , Gulley J , *et al*. A randomized phase II trial of docetaxel (taxotere) plus thalidomide in androgen‐independent prostate cancer. Semin Oncol 2001; 28 **:** 62–66. 10.1016/s0093-7754(01)90157-5 11685731

[45] Figg WD , Dahut W , Duray P , *et al*. A randomized phase II trial of thalidomide, an angiogenesis inhibitor, in patients with androgen‐independent prostate Cancer. Clin Cancer Res 2001; 7 **:** 1888–1893. 11448901

[46] Romero S , Stanton G , DeFelice J , *et al*. Phase II trial of thalidomide and daily oral dexamethasone for treatment of hormone refractory prostate cancer progressing after chemotherapy. Urol Oncol 2007; 25 **:** 284–290. 17628293 10.1016/j.urolonc.2006.09.017

[47] Hao L , Greer T , Page D , *et al*. In‐depth characterization and validation of human urine metabolomes reveal novel metabolic signatures of lower urinary tract symptoms. Sci Rep 2016; 6 **:** 30869. 27502322 10.1038/srep30869 PMC4977550

[48] Flevaris P , Vaughan D . The role of plasminogen activator inhibitor type‐1 in fibrosis. Semin Thromb Hemost 2017; 43 **:** 169–177. 27556351 10.1055/s-0036-1586228

[49] Bhattacharyya S , Wang W , Qin W , *et al*. TLR4‐dependent fibroblast activation drives persistent organ fibrosis in skin and lung. JCI Insight 2018; 3 **:** e98850. 29997297 10.1172/jci.insight.98850 PMC6124522

[50] Fielding CA , Jones GW , McLoughlin RM , *et al*. Interleukin‐6 signaling drives fibrosis in unresolved inflammation. Immunity 2014; 40 **:** 40–50. 24412616 10.1016/j.immuni.2013.10.022 PMC3919204

[51] Schafer S , Viswanathan S , Widjaja AA , *et al*. IL‐11 is a crucial determinant of cardiovascular fibrosis. Nature 2017; 552 **:** 110–115. 29160304 10.1038/nature24676 PMC5807082

[52] Uchinami H , Seki E , Brenner DA , *et al*. Loss of mMP 13 attenuates murine hepatic injury and fibrosis during cholestasis. Hepatology 2006; 44 **:** 420–429. 16871591 10.1002/hep.21268

[53] Dayan D , Hiss Y , Hirshberg A , *et al*. Are the polarization colors of picrosirius red‐stained collagen determined only by the diameter of the fibers? Histochemistry 1989; 93 **:** 27–29. 2482274 10.1007/BF00266843

[54] Junqueira LC , Cossermelli W , Brentani R . Differential staining of collagens type I, II and III by Sirius red and polarization microscopy. Arch Histol Jpn 1978; 41 **:** 267–274. 82432 10.1679/aohc1950.41.267

[55] Huang J , Zhang L , Wan D , *et al*. Extracellular matrix and its therapeutic potential for cancer treatment. Signal Transduct Target Ther 2021; 6 **:** 153. 33888679 10.1038/s41392-021-00544-0 PMC8062524

[56] Snedeker JG , Gautieri A . The role of collagen crosslinks in ageing and diabetes – the good, the bad, and the ugly. Muscles Ligaments Tendons J 2014; 4 **:** 303–308. 25489547 PMC4241420

[57] van der Slot‐Verhoeven AJ , van Dura EA , Attema J , *et al*. The type of collagen cross‐link determines the reversibility of experimental skin fibrosis. Biochim Biophys Acta 2005; 1740 **:** 60–67. 15878742 10.1016/j.bbadis.2005.02.007

[58] Boris A , Scott JW , DeMartino L , *et al*. Endocrine profile of a nonsteroidal antiandrogen *N*‐(3,5‐dimethyl‐4‐Isoxazolylmethyl)phthalimide (DIMP). Acta Endocrinol 1973; 72 **:** 604–614. 10.1530/acta.0.0720604 4739363

[59] Liu B , Su L , Geng J , *et al*. Developments in nonsteroidal antiandrogens targeting the androgen receptor. ChemMedChem 2010; 5 **:** 1651–1661. 20853390 10.1002/cmdc.201000259

[60] Nuttall FQ , Warrier RS , Gannon MC . Gynecomastia and drugs: a critical evaluation of the literature. Eur J Clin Pharmacol 2015; 71 **:** 569–578. 25827472 10.1007/s00228-015-1835-x PMC4412434

[61] Charest NJ , Zhou ZX , Lubahn DB , *et al*. A frameshift mutation destabilizes androgen receptor messenger RNA in the Tfm mouse. Mol Endocrinol 1991; 5 **:** 573–581. 1681426 10.1210/mend-5-4-573

[62] Maher TM , Strek ME . Antifibrotic therapy for idiopathic pulmonary fibrosis: time to treat. Respir Res 2019; 20 **:** 205. 31492155 10.1186/s12931-019-1161-4 PMC6731623

[63] Afari J , Spektor TM , Turner C , *et al*. Efficacy and safety of replacing lenalidomide with pomalidomide for patients with multiple myeloma refractory to a lenalidomide‐containing combination regimen. Exp Hematol 2022; 114 **:** 54–60. 35934183 10.1016/j.exphem.2022.07.303

[64] Davies FE , Leleu X , Vogel P , *et al*. A meta‐analysis of the efficacy of pomalidomide‐based regimens for the treatment of relapsed/refractory multiple myeloma after lenalidomide exposure. Clin Lymphoma Myeloma Leuk 2023; 23 **:** 829–837.e1. 37684184 10.1016/j.clml.2023.07.010

[65] Tamalunas A , Sauckel C , Ciotkowska A , *et al*. Inhibition of human prostate stromal cell growth and smooth muscle contraction by thalidomide: a novel remedy in LUTS? Prostate 2021; 81 **:** 377–389. 33687083 10.1002/pros.24114

[66] Tamalunas A , Sauckel C , Ciotkowska A , *et al*. Lenalidomide and pomalidomide inhibit growth of prostate stromal cells and human prostate smooth muscle contraction. Life Sci 2021; 281 **:** 119771. 34186051 10.1016/j.lfs.2021.119771

