# Supplementary material for: Targeting fibrosis in the treatment of lower urinary tract dysfunction
Source: J Pathol. 2026 May 27;269(4-5):493–503. doi: 10.1002/path.70079 (PMC13341276; doi:10.1002/path.70079)
Supplement: Supplementary file 1 — Figure S1. Thalidomide attenuates lower urinary tract dysfunction in aged mice Figure S2. Thalidomide does not lead to gross differences in inflammatory infiltrate in the mouse urogenital tract Table S1. Top differentially expressed genes in benign human prostate stromal cells treated with transforming growth factor β‐1 (TGFβ1) compared with vehicle Table S2. Top differentially expressed genes in benign human prostate stromal cells treated with transforming growth factor β‐1 (TGFβ1) + thalidomide compared with TGFβ1 Table S3. Forward and reverse primer sequences for housekeeping genes Table S4. Selected target amplicon context sequences for BioRad validated primers [file PATH-269-493-s001.docx]

**Targeting fibrosis in the treatment of lower urinary tract dysfunction**

AR Limkar *et al. J Pathol* <https://doi.org/10.1002/path.70079>

**Supplementary Figures S1 and S2**

**Supplementary Tables S1–S4**


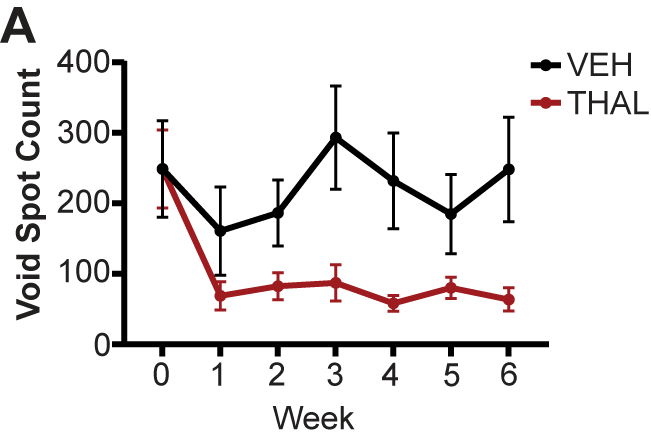


**Figure S1. Thalidomide attenuates lower urinary tract dysfunction in aged mice.** (A) Weekly void spot assay comparing vehicle (VEH) and thalidomide (THAL)-treated mice. Data are presented as mean±SEM.


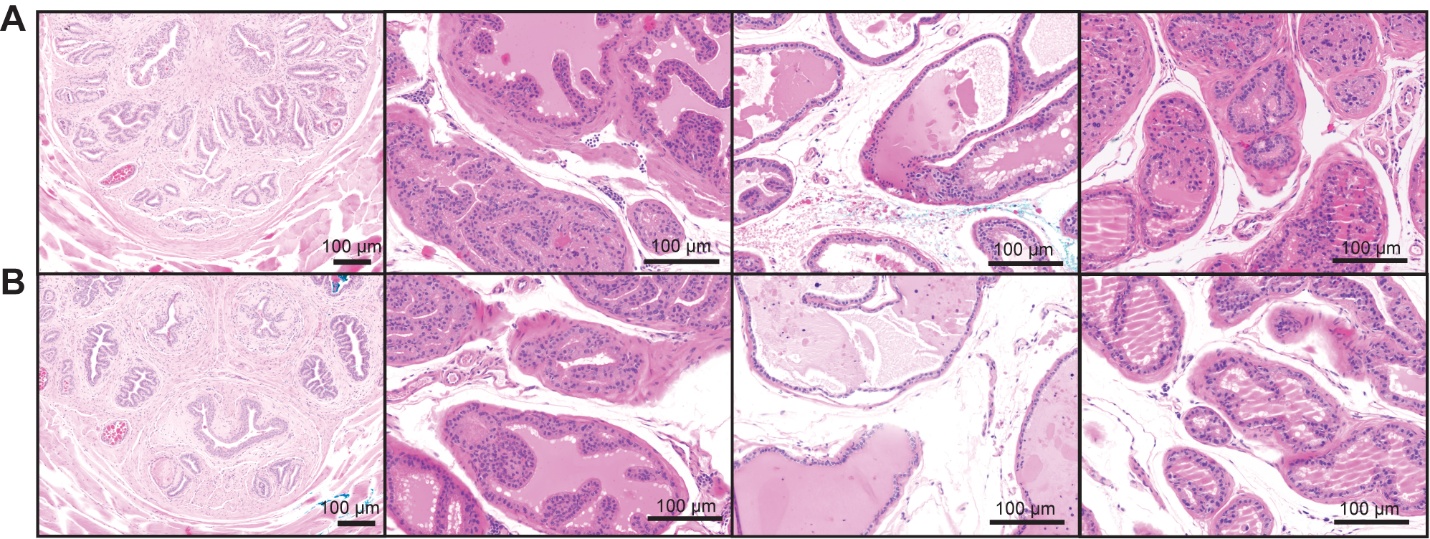


**Figure S2. Thalidomide does not lead to gross differences in inflammatory infiltrate in the mouse urogenital tract.** (A) From left to right, representative H&E images from vehicle-treated prostatic urethra, anterior prostate lobe, ventral prostate lobe, dorsolateral prostate lobe. (B) From left to right, representative H&E images from thalidomide-treated prostatic urethra, anterior prostate lobe, ventral prostate lobe, dorsolateral prostate lobe.

**Table S1.** Top differentially expressed genes in benign human prostate stromal cells treated with transforming growth factor β-1 (TGFβ1) compared with vehicle.

| Gene symbol | Log2(fold-change) | −Log10(*p* value) |
| --- | --- | --- |
| *CXCL8* | −2.95586 | 6.677781 |
| *VCAM1* | −2.90454 | 3.482052 |
| *ADH1B* | −2.44069 | 2.919622 |
| *CCL2* | −2.13067 | 4.872895 |
| *MASP1* | −1.74858 | 5.628932 |
| *ANGPTL4* | −1.55004 | 3.171953 |
| *ABCA1* | −1.41528 | 4.682982 |
| *COL14A1* | −1.24785 | 3.625307 |
| *RELB* | −1.06664 | 3.60401 |
| *IL33* | −1.04051 | 2.539001 |
| *LAMA3* | −0.9571 | 2.923148 |
| *IL6* | −0.95195 | 4.806319 |
| *IFI35* | −0.87211 | 2.859586 |
| *RAB7B* | −0.82618 | 2.626261 |
| *FABP4* | −0.78407 | 5.191789 |
| *SREBF1* | −0.75731 | 3.96465 |
| *MYLK* | −0.75233 | 4.835945 |
| *COL5A3* | −0.75212 | 2.497428 |
| *SCD* | −0.65437 | 5.005683 |
| *C1S* | −0.59499 | 4.357239 |
| *GNG2* | −0.5785 | 3.326362 |
| *CFH* | −0.57207 | 2.108986 |
| *SMAD3* | −0.56486 | 3.379968 |
| *FAS* | −0.56059 | 3.158615 |
| *HMOX1* | −0.54257 | 2.99552 |
| *COL5A1* | 1.361601 | 4.564315 |
| *MMP2* | 1.316865 | 6.080922 |
| *FN1* | 1.300087 | 4.364516 |
| *MMP13* | 1.284631 | 3.33613 |
| *MMP3* | 1.23229 | 4.665747 |
| *COL1A1* | 1.190561 | 3.814656 |
| *THBS1* | 1.153039 | 4.801068 |
| *MMP14* | 1.091991 | 6.49485 |
| *CYP7A1* | 1.041519 | 2.215161 |
| *HMGCS2* | 1.038045 | 2.55983 |
| *COL4A1* | 1.009994 | 4.142969 |
| *RPS6KA2* | 0.998113 | 2.084764 |
| *PTCH1* | 0.964547 | 2.649876 |
| *TLR4* | 0.953995 | 3.299054 |
| *PNOC* | 0.933736 | 2.924789 |
| *TTN* | 0.932753 | 2.22228 |
| *SERPINH1* | 0.882963 | 4.624336 |
| *COL4A2* | 0.872647 | 4.019043 |
| *COL1A2* | 0.868627 | 6.744727 |
| *PLPP4* | 0.862945 | 4.784891 |
| *COL7A1* | 0.832078 | 3.530399 |
| *FAP* | 0.798275 | 4.587539 |
| *TBC1D4* | 0.792301 | 3.429948 |
| *HSD11B1* | 0.77725 | 3.406659 |
| *PLCG2* | 0.747417 | 3.278585 |

**Table S2.** Top differentially expressed genes in benign human prostate stromal cells treated with transforming growth factor β-1 (TGFβ1) + thalidomide compared with TGFβ1.

| Gene symbol | Log2(fold-change) | −Log10(*p* value) |
| --- | --- | --- |
| *COL4A1* | −2.17874 | 5.826814 |
| *COL5A1* | −2.11716 | 7.69897 |
| *COL1A1* | −2.00588 | 4.492954 |
| *PLPP4* | −1.75127 | 6.721246 |
| *SERPINE1* | −1.71581 | 5.492144 |
| *THBS1* | −1.71164 | 4.551139 |
| *FN1* | −1.6942 | 5.739929 |
| *ITGA1* | −1.6789 | 8 |
| *IL11* | −1.5762 | 4.5223 |
| *COL7A1* | −1.55091 | 5.137869 |
| *COL4A2* | −1.32881 | 3.884623 |
| *IGF1R* | −1.32277 | 3.504414 |
| *COL1A2* | −1.31775 | 7.39794 |
| *ACTA2* | −1.28215 | 3.472306 |
| *TLR4* | −1.26106 | 3.623113 |
| *TGFB1* | −1.25329 | 4.724228 |
| *MMP2* | −1.23909 | 7.045757 |
| *LRP1* | −1.23883 | 4.377786 |
| *LOX* | −1.23395 | 4.563996 |
| *MMP13* | −1.20248 | 2.226956 |
| *PDGFRB* | −1.18639 | 3.087704 |
| *FAP* | −1.10718 | 7.09691 |
| *CDH2* | −1.09994 | 5.246417 |
| *ITGB3* | −1.05521 | 3.957661 |
| *TCF7L1* | −1.0015 | 4.058886 |
| *VCAM1* | 0.850374 | 2.095026 |
| *MMP1* | 0.777355 | 5.863279 |
| *CCL2* | 0.760325 | 3.038418 |
| *LAMA3* | 0.642988 | 2.11289 |
| *FABP5* | 0.493224 | 4.473015 |
| *SMAD3* | 0.490416 | 3.063818 |
| *EPAS1* | 0.417975 | 4.489723 |
| *FABP4* | 0.398898 | 3.137237 |
| *SLC25A10* | 0.387023 | 2.040733 |
| *SKP2* | 0.386524 | 2.277156 |
| *SCIN* | 0.37411 | 2.033859 |
| *PHLPP1* | 0.315818 | 3.561473 |
| *JAG1* | 0.292617 | 2.326891 |
| *HSP90AA1* | 0.277512 | 5.067526 |
| *ELOVL6* | 0.270061 | 2.115833 |
| *TXN* | 0.264732 | 4.453951 |
| *PSMB8* | 0.259292 | 3.318795 |
| *AP1S1* | 0.252084 | 3.030976 |
| *NID2* | 0.230279 | 2.205604 |
| *BCL2L1* | 0.217947 | 2.650157 |
| *HADH* | 0.208717 | 2.977423 |
| *AMOTL1* | 0.206483 | 3.594619 |
| *SEH1L* | 0.196793 | 5.127261 |
| *CAT* | 0.173363 | 2.340311 |
| *NCEH1* | 0.172955 | 2.952916 |

| **Table S3.** Forward and reverse primer sequences for housekeeping genes. | | |
| --- | --- | --- |
| Gene symbol | Primer direction | Primer sequence |
| *TBP* | Forward | CCACTCACAGACTCTCACAAC |
|  | Reverse | CTGCGGTACAATCCCAGAACT |
| *YWHAZ* | Forward | TGATCCCCAATGCTTCACAAG |
|  | Reverse | GCCAAGGTAACGGTAGTAATCT |

| **Table S4.** Selected target amplicon context sequences for BioRad validated primers. | |
| --- | --- |
| Gene symbol | Amplicon context sequence |
| *COL1A1* | TCTTGGTCTCGTCACAGATCACGTCATCGCACAACACCTTGCCGTTGTCGCAGA CGCAGATCCGGCAGGGCTCGGGTTTCCACACGTCTCGGTCATGGTACCTGAGG CCGTTCTGTACGCAGGTGATTGGTGGGATGTCTTCG |
| *COL3A1* | ACACCGATGAGATTATGACTTCACTCAAGTCTGTTAATGGACAAATAGAAAGCCT CATTAGTCCTGATGGTTCTCGTAAAAACCCCGCTAGAAACTGCAGAGACCTGAAA TTCTGCCATCCTGAACTCAAGAGTGGA |
| *COL5A1* | AAGAGATCTTCGGCTCTCTCAACTCTCTGAAGCTGGAGATTGAGCAGATGAAAC GGCCCCTGGGCACGCAGCAGAACCCCGCCCGCACCTGCAAGGACCTGCAGCT CTGCCACCCCGACTTCCCAGATGGTGAATACTGGGTCGATCCTAACCAAGGATG CTCCAGGGATTCCTTCAAGGTTTACTGCAACTTCACAGCCGGGGGGTCGACATG CGTCTTCCC |
